# Supplementation of Culture Media with Lysophosphatidic Acid Improves The Follicular Development of Human Ovarian Tissue after Xenotransplantaion into The Back Muscle of γ-Irradiated Mice 

**DOI:** 10.22074/cellj.2020.6752

**Published:** 2019-12-15

**Authors:** Zeynab Mohammadi, Nasim Hayati Roodbari, Kazem Parivar, Mojdeh Salehnia

**Affiliations:** 1 Department of Biology, Science and Research Branch, Islamic Azad University, Tehran, Iran; 2 Department of Anatomy, Faculty of Medical Sciences, Tarbiat Modares University, Tehran, Iran

**Keywords:** Apoptosis, Lysophosphatidic Acid, Ovarian Follicle

## Abstract

**Objective:**

The aim of the present study was to evaluate the effects of lysophosphatidic acid (LPA) supplementation of human ovarian tissue culture media on tissue survival, follicular development and expression of apoptotic genes following xenotransplantation.

**Materials and Methods:**

In this experimental study, human ovarian tissue was collected from eight normal female to male transsexual individuals and cut into small fragments. These fragments were vitrified-warmed and cultured for 24 hours in the presence or absence of LPA, then xenografted into back muscles of γ-irradiated mice. Two weeks post-transplantation the morphology of the recovered tissues were evaluated by hematoxylin and eosin staining. The expression of genes related to apoptosis (*BAX* and *BCL2*) were analyzed by real time revers transcription polymerase chain reaction (RT-PCR) and detection of BAX protein was done by immunohistochemical staining.

**Results:**

The percent of normal and growing follicles were significantly increased in both grafted groups in comparison to the non-grafted groups, however, these rates were higher in the LPA-treated group than the non-treated group (P<0.05). There was a higher expression of the anti-apoptotic gene, BCL2, but a lower expression of the pro-apoptotic gene, BAX, and a significant lower BAX/ BCL2 ratio in the LPA-treated group in comparison with non-treated control group (P<0.05). No immunostaining positive cells for BAX were observed in the follicles and oocytes in both transplanted ovarian groups.

**Conclusion:**

Supplementation of human ovarian tissue culture medium with LPA improves follicular survival and development by promoting an anti-apoptotic balance in transcription of *BCL2* and *BAX* genes.

## Introduction

Fertility preservation by using cryopreserved ovarian tissue is critical for patients who are subjected to chemotherapy and radiotherapy or suffer from premature ovarian failure and autoimmune problems (1). The ovarian tissue is cryopreserved by two slow freezing and vitrification techniques. Based on the literature vitrification may be more effective than slow freezing, based on less primordial follicular DNA damage and better preservation of stromal cells (2). *In vitro* culture (IVC) followed by transplantation of cortical ovarian tissue is a potential technique to develop and grow the follicles after cryopreservation. The results obtained from these techniques in human ovarian tissue are very controversial due to their large sizes, dense ovarian stroma and long folliculogenesis period (3-4).

Apoptosis that is induced by oxidative stress or physical and chemical triggers during IVC and transplantation in cryopreserved tissues, affects the quality, growth, survival, and development of ovarian follicles (5-9). The usage of appropriate growth factors, antioxidants and anti-apoptotic factors improves the quality of the tissue during both IVC and grafting procedure (10-13).

Lysophosphatidic acid (LPA) is a bioactive phospholipid that is present in all tissues and plays roles in several cell activities such as proliferation, differentiation and migration (14,15). In ovaries and uterus LPA signaling is involved in early embryo development and preparation of endometrium for embryo-maternal interactions (14,16- 21). LPA and its active receptors have been reported to be expressed in uterus, ovaries, and placenta (15, 16). Recent studies on several mammalian species showed that LPA does its function through interactions of its LPAR1-6 receptors (16-22). Out of the six LPA receptors, LPAR4 is highly expressed in the cortex of human ovaries and LPAR1- 3 are detected in human granulosa-lutein cells (15).

In addition, the effects of LPA as an anti-apoptotic factor on several cell types have been suggested in the literature (17, 21-23). However, there is poor information regarding its effects for improving the cell quality in IVC of human ovarian tissue. Therefore, the aim of the present study was to evaluate the effects of supplementation of the human ovarian tissue culture media with LPA on tissue survival and follicular development after xenotransplantation, using morphological and immunohistochemical techniques as well as analysis of the expression of apoptosis-related genes.

## Materials and Methods

All reagents used in the following experiments were obtained from Sigma-Aldrich (Germany), unless stated otherwise.

### Ovarian tissue collection

In this experimental study, the human ovarian tissues were collected from 8 normal transsexual (female to male) individuals aged 18-35 years old (median 26.1). The tissues were received following laparoscopic surgery under confirmation by Ethics Committee of the Faculty of Medical Science of Tarbiat Modares University (Ref. No. 52/883). The ovarian cortical tissues were cut into approximately 2×1×1 mm pieces under sterile conditions (n=130). These fragments vitrified-warmed and all assessments of this study were performed on these samples. All samples were cryopreserved and stored at liquid nitrogen until they were used.

### Experimental design

This experimental study was designed to assess the effect of LPA on human ovarian tissue morphology and expression of some apoptosis-related genes after xenotransplantation. After vitrification and warming of ovarian fragments, the tissues were cultured 24 hours in the presence or absence of LPA, then xenotransplanted into gluteus maximus muscles of γ-irradiated female NMRI mice. Before and after transplantation tissue morphology and follicular counting were assessed by hematoxylin and eosin (H&E) staining. Analysis of expression of the apoptosis-related genes (*BAX* and *BCL2*) was performed by real time revers transcription polymerase chain reaction (RT-PCR). Also immunohistochemical staining for BAX protein was done on recovered transplanted tissue.

### Ovarian tissue vitrification and warming

The ovarian cortical fragments (n=125) were vitrified according to the protocol described previously in the solution ethylene glycol, ficol and sucrose named: EFS40% (6). The human ovarian tissues were equilibrated in three changes of vitrification solutions, then they were put into cryovials and stored in liquid nitrogen. For warming the ovarian tissues they were hydrated by serially diluted sucrose (1, 0.5 and 0.25 M phosphate buffer) and equilibrated with culture media for 30 minutes for the following assessments. Some ovarian fragments (n=5 fragments) were fixed in Bouin’s solution for evaluation of normal morphology after warming and the other fragments were used for *in vitro* culturing (n=120 fragments).

### Ovarian tissue culture

Vitrified-warmed tissue fragments were cultured (n=120 fragments in total) in multi-well culture plates, containing 300 μl/well of α-MEM supplemented with 5 mg/ml human serum albumin (HSA), 0.1 mg/ml penicillin G, 0.1 mg/ml streptomycin, 10 μg/ml insulintransferrin-selenium, 0.5 IU/ml human recombinant follicle stimulating hormone, with or without 20 μM LPA at 37˚C in humidified chamber with 5% CO_2_ (24). Some of these cultured ovarian fragments were used for histological evaluation, follicular counting and molecular analysis before transplantation (n=30 for each group) and the others were transplanted into γ-irradiated mice (n=30 for each group).

### γ-irradiated mice preparation and xenotransplantation of human ovarian tissue

The 8-weeks-old NMRI female mice (n=30 mice for each group) were each given a single dose of 7.5 Gy whole body γ-irradiation for 6 minutes (Theratron 780C, Canada). For human ovarian tissue transplantation, the mice were anesthetized by an intra-peritoneal injection of ketamine 10% (75 mg/kg body weight) and xylazine 2% (15 mg/kg) and their back muscles were bilaterally exposed (25). Each tissue fragment that was derived from either LPA-treated or non-treated groups was individually inserted and stitched within each muscle (two ovarian fragments for each mouse), and the wound was sutured. The mice were sacrificed 14 days after transplantation and the recovered tissues were randomly fixed for histological and immunohistochemical analyses (n=15 tissue fragments for each group) or kept at -80˚C for molecular studies (n=15 tissue fragments in each group for triplicates).

### Histological evaluation

For the light microscopic study, the fresh (n=5 fragment), vitrified-warmed (n=5 fragment), LPA-treated and nontreated human ovarian fragments before (n=15 fragments for each group) and after transplantation (n=15 fragments for each group) were fixed in Bouin’s solution and embedded in paraffin wax. Tissue sections were prepared serially at 5 μm thickness and every 10th section was stained with H&E and observed under a light microscope (near 15-20 sections per each fragment). Another set of tissue sections was prepared for immunohistochemistry.

The follicle classification criteria included: those containing an intact oocyte as well as granulosa cells (normal), those containing pyknotic oocyte nuclei or disorganized granulosa cells (degenerated), those containing only a single layer of flattened granulosa cells (primordial), those with cuboidal granulosa cells in a single layer (primary), and finally those with two or more layers of granulosa cells (growing follicles).

### Immunohistochemical staining for BAX

The expression of pro-apoptotic protein BAX in transplanted LPA-treated and non-treated ovarian tissue was confirmed by immunohistochemistry. After paraffin removal, antigen retrieval was performed by boiling the tissue sections in citrate buffer (10 mM, pH=6) in a microwave oven for 10 minutes at 700 W. Then they were cooled at room temperature and washed in phosphate buffer saline (PBS). The tissue sections were separately incubated in rabbit polyclonal immunoglobulin G (IgG) anti-BAX antibody (SC-493, 1: 100) (Santa Cruz Biotechnology, UK) at 4˚C overnight, then were washed three times in PBS. Then they were incubated with a secondary goat anti-rabbit IgG antibody conjugated with fluorescein isothiocyanate (FITC) (Ab 6721, 1:100, Abcam, UK) diluted in PBS for 2 hours at 37˚C. Tissue sections from adult mouse ovaries were used as positive controls and were stained according to the same protocol. The samples were analyzed under fluorescent microscope (Ziess, Germany).

### RNA extraction and cDNA synthesis

Total RNA was extracted from LPA-treated and nontreated ovarian tissue fragments before (n=15 in each group in three repeats) and after (n=15 in each group in three repeats) grafting, using Trizol reagent (Invitrogen, UK) and according to the manufacturer’s protocol. The RNA samples were treated with DNase prior to proceeding with the cDNA synthesis. RNA concentration was measured by spectrophotometry. The cDNA synthesis was performed using a commercial Kit (Thermo Scientific, EU) at 42˚C for 60 minutes and the reaction was terminated by heating the samples at 70˚C for 5 minutes. The obtained cDNA was stored at -80˚C until utilized.

### Real-time revers transcription polymerase chain reaction

Primers for the apoptosis-related genes, *BAX* and *BCL2*, and housekeeping *β-actin* (Table 1) were designed using the online primer3 software. One-step RT-PCR was performed on the Applied Biosystems (UK) real-time thermal cycler according to QuantiTect SYBR Green RTPCR kit (Applied Biosystems, UK, Lot No: 1201416). Real-time thermal cycling conditions were set up as follows: holding step at 95˚C for 5 minutes, cycling step at 95˚C for 15 seconds, 60˚C for 30 seconds and it was continued by a melt curve step at 95˚C for 15 seconds, 60˚C for 1 minutes, and 95˚C for 15 seconds. Then, relative quantification of the target genes to housekeeping gene (*β-actin*) was determined by Pfaffl method. The nontemplate negative control sample was included in each run. These experiments were repeated at least three times.

### Statistical analysis

All experiments were repeated in triplicates. All data were presented as mean ± SD and were analyzed, using one-way ANOVA and post hoc Duncan’s Multiple Range Test. Statistical analysis was performed with the SPSS 19.0 (Chicago, IL, USA). A P<0.05 was considered statistically significant.

## Results

### Histological observation

**Fig 1:**
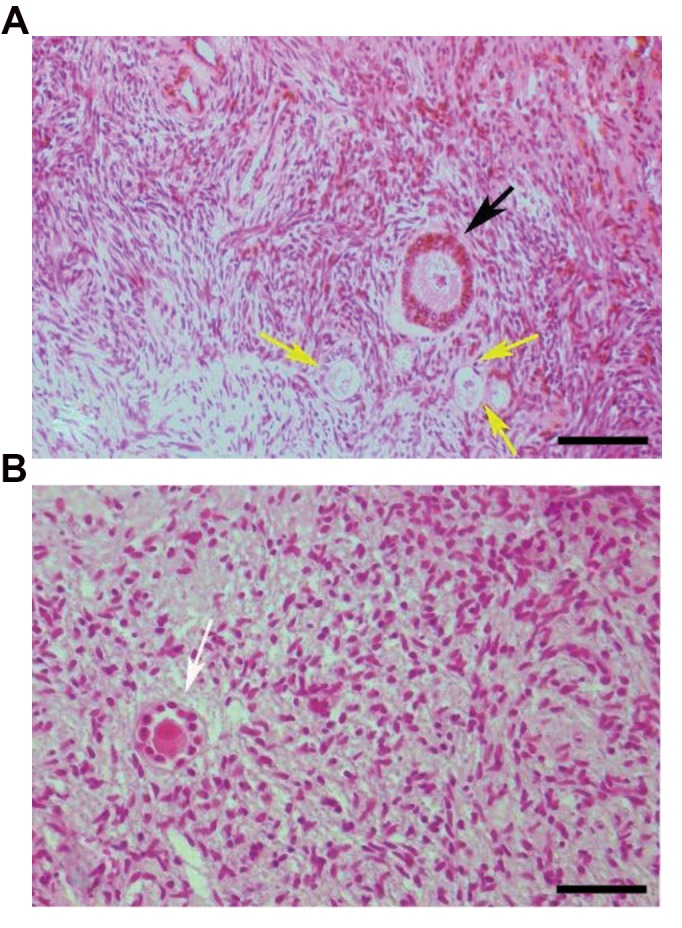
Light microscopic observation of fresh and vitrified human ovarian tissue using hematoxylin and eosin. **A.** The morphology of human ovarian tissue were demonstrated in fresh (scale bar: 60 µm) and **B.** Vitrifiedwarmed groups (scale bar: 30 µm). The normal primordial (yellow arrows), primary (white arrow) and growing follicles (black arrow) are seen.

The normal morphology of human ovarian tissue after vitrification-warming in comparison to a fresh sample is presented in Figure 1 A and B. As shown in this Figure, the structure of tissue is cryopreserved well and no significant damage is seen in the follicles or stromal cells.

**Table 1:** The characteristics of primers used for the the real time revers transcription polymerase chain reaction


Target gene	Primer sequence (5´-3´)	Accession number	Product size (bp)

**BCL2**	F: TTGCTTTACGTGGCCTGTTTC	NM_000018.9	94
	R: GAAGACCCTGAAGGACAGCCAT		
**BAX**	F: CCCGAGAGGTCTTTTTCCGAG	NM_000019.9	155
	R: CCAGCCCATGATGGTTCTGAT		
**β-actin**	F: TCAGAGCAAGAGAGGCATCC	NM_001101.3	187
	R: GGTCATCTTCTCACGGTTGG		


**Fig 2:**
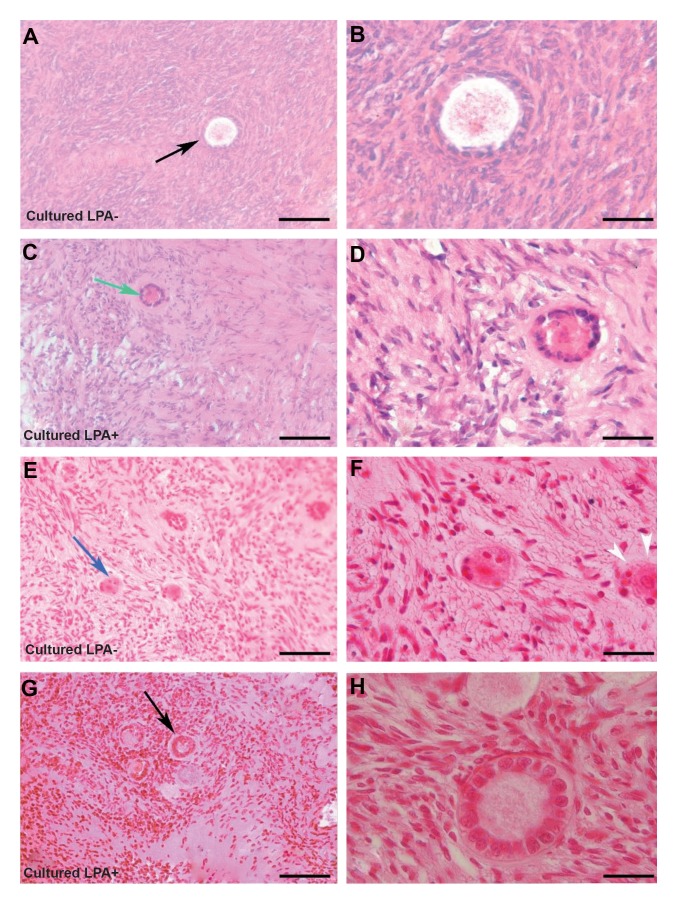
Light microscopic images of the cultured human ovarian tissue and xenografted tissue using hematoxylin and eosin staining. The micrograph of cultured tissues before transplantation **A, B.** In non-treated group and **C, D.** In LPA-treated group. The morphology of tissues after transplantation **E, F.** In non-treated group and **G, H.** In LPA-treated group. The images in the second panel are showing high magnifications of the first panel. The morphology of normal primordial follicles (blue arrows), primary follicle (green arrow) and growing follicle (black arrow) are shown. The white arrow head shows detachment of the follicular cells in the non-treated group after grafting (scale bar: A, C, E, G: 30 µm, B, D, F, H: 20 µm).

The light microscopic observations of LPA-treated and non-treated human ovarian fragments after 24 hours of IVC are illustrated in Figure 2 A-D. The normal morphology of the growing follicles with central oocytes are seen and the oocytes are in close contact with the surrounding granulosa cells. Two weeks after grafting, the primordial, primary and growing follicles are detected in tissue sections (Fig .2E-H), however, the detachment between the oocyte and granulosa cells are observed in some follicles in non-treated grafted ovarian sections (Fig .2F).

### The percent of normal follicles in the study groups

The proportion of the follicles at different developmental stages in our study groups is summarized in Table 2. After 24 hours into the cultures of ovarian fragments, the number of normal follicles in the LPAtreated group is significantly higher than those in the not-treated group [88.01 ± 2.62% vs. 81.72 ± 2.31% (P<0.05)]. Moreover, 14 days after transplantation, in the LPA-treated group 91.62 ± 0.70% of the follicles presented normal morphology, which was significantly higher (P<0.05) than that in the non-treated group (87.97 ± 1.61%).

### The percentage of follicles at different developmental stages in study groups

The proportion of the follicles at different developmental stages in all experimental groups are compared and presented in Table 2. The percentage of the primordial follicles in non-treated cultures as well as LPA-treated groups prior to transplantation are 41.78 ± 4.61% and 42.49 ± 1.13%, respectively. Following transplantation the non-treated and treated groups decline significantly to 30.46 ± 6.86 and 21- 17 ± 6.01, respectively (P<0.05). However, this posttransplantation percentage is significantly lower in the LPA-treated group compared to the non-treated group (P<0.05). There is no significant difference between the percentages of the primary follicles in the two study groups (Table 2).

The total percentages of the growing follicles in the LPA-treated group and the non-treated group prior to transplantation are 20.17 ± 2.39 and 19.49 ± 1.65, and are increased after transplantation to 40.95 ± 2.11 and 29.44 ± 1.39, respectively (P<0.05). This difference is significantly higher in the LPA-treated group compared to the non-treated group (P<0.05, Table 2).

### Immunohistochemistry

**Fig 3:**
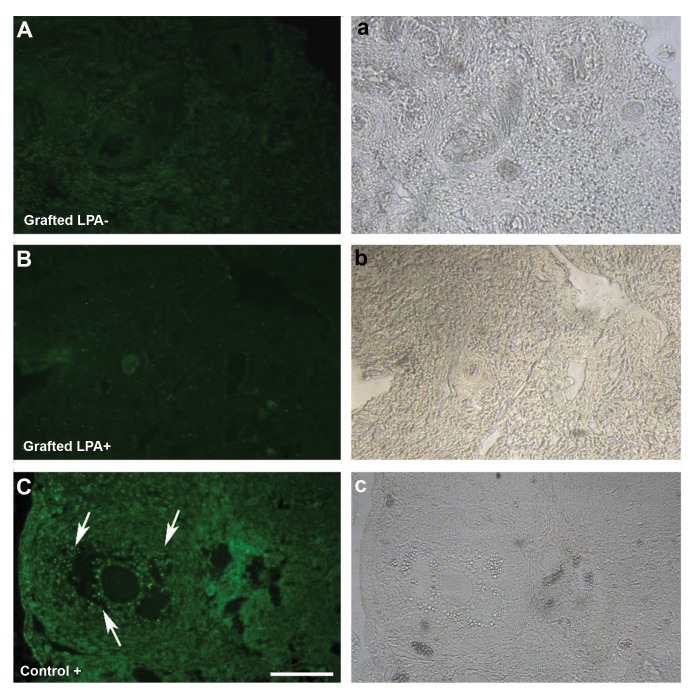
The images of immunohistochemistry of BAX in studied groups. Representative figures of immunostained cells **A, a.** In non-treated group, **B, b.** LPA-treated group after transplantation were demonstrated, and C, c. Adult mouse ovarian tissue served as the positive control. White arrows show the BAX-positive cells. The left panel show the phase contrast of the images in the right panel (scale bar: 100 µm). LPA; Lysophosphatidic acid.

The representative images of BAX immunohistochemistry in both transplanted ovarian tissue and positive tissue section as control are shown in Figure 3A-C. In spite of the presence of several BAX-positive cells (white arrow) in the follicular and stromal cells of the adult mouse ovarian tissue as positive control (Fig .3C), no other positive labeling for BAX was observed in the follicles and oocytes in neither transplanted ovarian group.

### Expression of apoptosis-related genes in studied groups

**Fig 4:**
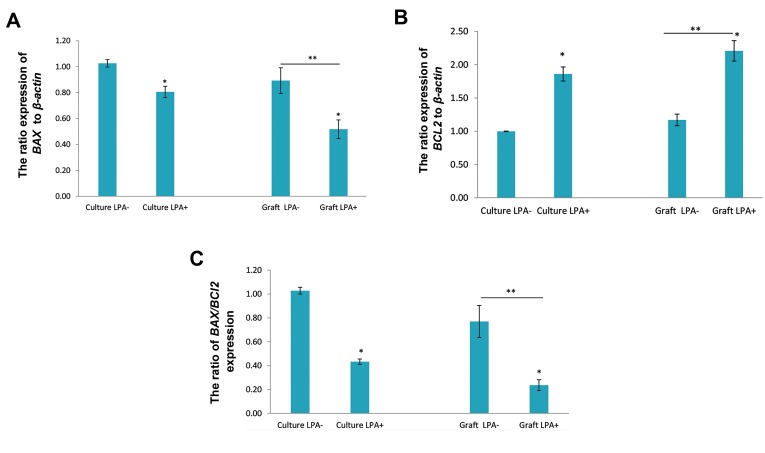
The comparison of the expression ratio of pro-apoptotic and antiapoptotic genes in studied group. The expression of **A.** *BAX* and **B.** *BCl2* genes to the housekeeping gene (*β-actin*) and the ratio of BAX/BCL2 expression were presented. LPA; Lysophosphatidic acid, *; Significant difference with the non-treated groups (LPA) (P<0.05), and **; Significant differences of both transplanted groups with respected non-transplanted groups (P<0.05).

The expression ratio of **BAX** and **BCL2** genes to the housekeeping gene (**β-actin**) in both study groups is shown in Figure 4. Our results indicate that, the expression ratio of the **BAX** gene in the LPA-treated group is significantly lower than that in the nontreated group (P<0.05) both before and after grafting. Nonetheless, the level of **BCL2** gene expression is significantly higher in the LPA-treated group compared to the non-treated ovarian tissue (P<0.05) before grafting (Fig .4A, B) also the same result was obtained after grafting (P<0.05). Also, the ratio of **BAX** to **BCL2** expression in the LPA-treated group is significantly less than that in the non-treated ovarian tissue (P<0.05, Fig .4C).

**Table 2:** The number of follicles at different developmental stages in all groups of study


Groups(vitrified-ovarian tissue)	Total number of F.	Number of normal F.	Number of degenerated F.	Number of primordial F.	Number of primary F.	Number of growing F.

Cultured-LPA^-^	90	73 (81.72 ± 2.31)	17 (18.28 ± 0.99)	30 (41.78 ± 4.61)	29 (38.73 ± 4.68)	14 (19.49 ±1.65)
Cultured-LPA^+^	134	119(88.01 ± 2.62)^a^	15 (11.99 ± 2.62)^a^	51 (42.49 ± 1.13)	43 (37.34 ± 3.46)	25 (20.17 ± 2.39)
Cultured-grafted-LPA^-^	258	227(87.92 ± 1.61)^a^	31 (12.08 ± 1.61)^a^	71 (30.46 ± 6.86)^a^	90 (40.46 ± 7.49)	67 (29.44 ± 1.39)^a^
Cultured-grafted-LPA^+^	223	204 (91.62 ± 0.70)^b^^,^^c^	19 (8.38 ± 0.70)^b^^,^^c^	41 (21.17 ± 6.01)^c^	80 (38.44 ± 4.40)	84 (40.95 ± 2.11)^b^^,^^c^


LPA; Lysophosphatidic acid, F; Follicle, ^a^; Significant difference with cultured-LPA- (without LPA) group in the same column (P<0.05), ^b^; Significant differences with cultured-LPA+ (with LPA) group in the same column (P<0.05), and ^c^; Significant differences with cultured-grafted- LPA- (without LPA) group in the same column (P<0.05). The number of follicles at different developmental stages was calculated according to the total number of normal follicles. Data are presented as %mean ± SD.

## Discussion

This is to our knowledge the first report to evaluate the effect of LPA on the improvement of development and survival of human ovarian follicles after IVC as well as transplantation of ovarian tissue. Our morphological observations indicate an enhancement in the rate of normal follicles and a decrease in the percentage of degenerated follicles in the LPA-treated group in comparison to the non-treated group. This result shows the beneficial effects of LPA on the preservation of the follicles within the human ovarian tissues during an IVC period and following transplantation. These effects of LPA may be related to its function as an anti-apoptotic factor (17, 21- 23). Apoptosis take places within the ovarian cells through two main pathways, including the activation of caspase 8, and the mitochondrial pathway that is controlled by BAX and BCL2 as regulatory proteins (26-28). In agreement with our morphological analysis, immunohistochemical staining showed very low number of BAX positive cells in the transplanted groups, especially in the LPA-treated group. According to the literature, the anti-apoptotic effects of LPA on oocyte, granulosa cells, ovarian cancer cells and corpus luteum are documented (17, 21, 23, 27). In the study by Rapizzi et al. (29) it was shown that LPA induced migration and survival in the cervical cancer cells line, HeLa cells. Similarly, in the bovine corpus luteum, it was demonstrated that LPA inhibited the expression of BAX, therefore contributing to the survival of the cells (23). Sinderewicz et al. (19) and Boruszewska et al. (30) demonstrated that in healthy bovine follicles, LPA interacts with estradiol to stimulate the anti-apoptotic processes of granulosa cells.

In addition, molecular analysis in the present study revealed a significantly higher expression of *BCL2* and lower expression of *BAX* in the LPA-treated group in comparison with the non-treated group. Moreover, we found a significantly lower* BAX/BCL2* ratio in the LPA-treated group compared to the non-treated ones. As *BCL2* and *BAX* have been detected in the granulosa cells, it has been suggested that follicular viability and development may depend on a low level of pro-apoptotic gene expression, which prevents cell death within ovarian tissue.

In agreement to our observations, in the study by Zhang et al. (18) the authors have shown that exposure of blastocyst culture media to LPA reduces the expression of the pro-apoptotic genes, while increasings the expression of anti-apoptotic genes. Similar results were obtained by Boruszewska et al. (17) in their study on bovine oocyte.

Our current data demonstrates that LPA could enhance the follicular growth and development, as the culture media used in our study seems to support the activation and development of growing follicles. The growth of follicles depends mainly on proliferation rate of the granulosa cells. It is proposed that LPA could be involved in proliferation and growth of the follicles directly via its receptors, or indirectly by stimulation of some other factors (16-22). In agreement with these suggestions, it has been previously revealed that in the mitogenic effects of LPA on ovarian, tumor, and amniotic cells, mitogenactivated protein kinase (MAPK)/p38 and phosphoinositol 3-kinase (PI3K)/Akt pathways are involved (31-33). Kim et al. (31) also have found that LPA modulates cellular activity and stimulates proliferation of human amnion cells *in vitro*. These authors also proposed that the LPA produced in leiomyoma may be involved in tumor cell proliferation.

With regard of another suggestion that was well demonstrated previously by Boruszewska et al. (30), it is possible that LPA alone or LPA together with follicle stimulating hormone induced estradiol (E2) are byproducts of *in vitro* cultures of bovine granulosa cells. Thus, the secretion of these hormones causes an increase in the expression of the follicle stimulating hormone receptor and 17β –Hydroxysteroid dehydrogenase (*HSD*) genes that are involved in follicular growth and development. Our results are in agreement with that reported by Abedpour et al. (22, 24), who stated that LPA can improve the developmental and maturational rates of the follicles in cultured mouse ovarian tissue. Related reports show that LPA plays a significant role in activation of the primordial follicles and improves nuclear and cytoplasmic maturation of mouse oocytes via its receptors (33). In 2015 Zhang et al. (18) performed a similar study and demonstrated that LPA had beneficial effects on porcine cytoplasmic oocyte maturation. The work by Boruszewska et al. (17) also revealed that supplementation of bovine oocyte maturation media with LPA increased expression of some oocyte developmental genes such as growth differentiation factor 9 (GDF9) and follistatin (FST) transcripts. Hwang et al. (34) by treatment of porcine oocytes during *in vitro *maturation with different concentrations of LPA showed that 30 μM LPA promotes and enhances cumulus cell expansion and oocyte nuclear and cytoplasmic maturation, and reduces the intracellular reactive oxygen species level.

In contrast to our current report, in our previous study we had grafted the vitrified human ovarian tissue, and the rate of normal follicles was significantly decreased in the vitrified grafted tissues. In the present study, however, the tissue was cultured for 24 hours prior to transplantation. It is suggested that during the time of cultivation, especially in the presence of LPA, the harmful effects of cryopreservation are recovered to some extent. In a published study by Rahimi et al. (35), similar to our groups, they observed a higher incidence of apoptosis in grafted vitrified ovarian tissue samples without any supplementary factors added to the transplanted tissue. To prove this suggestion additional assessments are needs.

Moreover, our observations showed the percentage of normal follicles was higher in both transplanted groups compared to their respected non-transplanted tissues at the end of the culture period. An explanation for this result is that in spite of degeneration of some follicles due to ischemia in the grafted tissue, these damaged follicles were disappeared during these two weeks following engraftment. It seems that the total number of the follicles may decline per each tissue section (was not calculated), while we have analyzed the ratio of normal follicles in comparison to the total number of the counted ones.

## Conclusion

Supplementation of human ovarian tissue culture media with LPA could improve the follicular survival and development by promoting an anti-apoptotic balance in transcription of *BCL2* and *BAX* genes, leading to increased cell survival.
